# Differentiating malignant from benign thyroid nodules with indeterminate cytology by ^99m^Tc-MIBI scan: a new quantitative method for improving diagnostic accuracy

**DOI:** 10.1038/s41598-017-06603-3

**Published:** 2017-07-21

**Authors:** A. Campennì, M. Siracusa, R. M. Ruggeri, R. Laudicella, S. A. Pignata, S. Baldari, L. Giovanella

**Affiliations:** 1 Department of Biomedical and Dental Sciences and Morpho-functional Imaging, Nuclear Medicine Unit, University of Messina, Messina, Italy; 2 Department of Clinical and Experimental Medicine, Unit of Endocrinology, University of Messina, Messina, Italy; 3 Department of Nuclear Medicine and Thyroid Centre, Oncology Institute of Southern Switzerland, Bellinzona, Switzerland

## Abstract

Quantitative ^99m^Tc-MIBI thyroid scintigraphy is a useful tool in differentiating malignant from benign thyroid nodules with indeterminate cytology. The aim of our report is to compare the diagnostic performance of different quantitative methods. We prospectively evaluated 20 patients affected by a thyroid nodule with a cytological diagnosis of class III or IV according to the Bethesda system. Planar images of the thyroid were acquired 10 and 60 minutes after ^99m^Tc-MIBI administration and two different quantitative methods applied (i.e. wash-out index, WO*ind;* retention index, R.I.). All patients underwent lobectomy or thyroidectomy and final histological findings were matched with MIBI results obtained with both quantitative methods. Four out of 20 patients had a final histological result of differentiated thyroid cancer, while benign findings were found in the remaining cases. Overall sensitivity, specificity, accuracy, PPV and NPV were 100% in all for the WO*ind* and 100%, 57.1%, 62.5%, 25% for the R.I., respectively. In conclusion ^99m^Tc-semiquantitative MIBI thyroid scintigraphy with WO*ind* calculation is highly accurate in differential diagnosis of nodules with indeterminate cytology reading.

## Introduction

Thyroid nodular disease is a worldwide clinical problem and, according to the method of detection, prevalence ranges from 20–50% of the general population^1^ with higher prevalence in currently and previously iodine-deficient areas. However, thyroid cancers are rare, accounting for about 2–5% of all thyroid nodules^2–4^. Diagnostic evaluation of thyroid nodules includes laboratory tests and thyroid ultrasonography (US). Additionally, thyroid scintigraphy, with either 123-radioiodine or ^99m^Tc-pertechnetate, is performed to disclose autonomously functioning nodules that are classified as benign, with a high degree of certainty. US features are also employed to stratify the risk of malignancy and to select those nodules requiring US-guided fine needle aspiration cytology (FNAC)^5, 6^. This diagnostic algorithm has proven to be accurate in detecting or excluding thyroid cancer. However, faced with follicular-patterned lesions, cytopathologists cannot accurately discriminate benign from malignant lesions, since detection or exclusion of capsular and/or vascular invasion cannot be done on cytological specimens^7, 8^. As the large majority of such lesions are benign, the risk of inappropriate thyroid surgery is significant. In order to improve accuracy of cytological diagnosis, two main classification systems are now used in daily practice^9, 10^. However, the number of patients who actually have a benign lesion at histological evaluation remains quite high (55–85%)^10–14^. Some Authors have already reported on the role of 99mTc-MIBI in thyroid disease^15^ and in particular in differentiating malignant from benign lesions in patients with non-diagnostic/indeterminate cytology^5, 16–19^. At visual evaluation (i.e. qualitative analysis), a negative MIBI scan (i.e. absent uptake in nodule) has a very high negative predictive value (NPV) in excluding thyroid malignancies^5, 16, 19, 20^ while a positive MIBI scan (i.e. MIBI nodular uptake >^99m^Tc-pertechnetate on early image; MIBI nodular uptake ≥MIBI parenchyma uptake) can be found both in malignant and benign lesions^5, 16–18^. Thus, the specificity and positive predictive value (PPV) of MIBI scan are always quite low in these patient settings^5, 18^. Quantitative analysis of MIBI thyroid scan has proven to increase diagnostic accuracy^21^. Saggiorato^19^ first evaluated the tracer Retention Index (R.I.) by using a semi-quantitative method based on the ratio between uptake within the nodule and the normal thyroid tissue uptake. More recently, Giovanella *et al*.^7^ and Campenni’ *et al*.^22^ evaluated the tracer Wash-Out Index (WO*ind*) from the nodule while uptake within normal thyroid tissue was not taken into account. This is a crucial difference between the two methods, R.I and WO*ind*, since the MIBI wash-out from normal parenchyma is faster than MIBI wash-out from nodules. The aim of the present technical note is to compare these two methods for quantitative MIBI uptake analysis in a series of patients harboring cytologically indeterminate thyroid nodules.

## Material and Methods

### Patients

We prospectively evaluated 20 patients (F = 17, M = 3; mean age 50.4 ± 16.6, range 20–80 years, median age: 51 years; F/M ratio = 5.6:1) affected by nodular thyroid goitre and referred to the Nuclear Medicine and Endocrine Units at “G. Martino” University Hospital, Messina (Italy), from January 1, 2015 through February 29, 2016. All patients fulfilled the following inclusion criteria: (a) age ≥18 years; (b) TSH levels >0.40 mUI/L and calcitonin values <5 ng/L (women) and 10 ng/l (men); (c) one solid thyroid nodule, ≥15 (mm) in maximum diameter, solid at US and cold at ^99m^Tc-pertechnetate scintigraphy and, (d) a cytological diagnosis of class III or IV according to the Bethesda system. All patients underwent thyroid surgery (near)-total thyroidectomy in order to confirm or exclude a malignant lesion. Before surgery patients underwent ^99m^Tc-MIBI thyroid scintigraphy and finally, scintigraphic results were compared to postoperative histology findings.

### Ethics

The study was approved by the Ethics Committee of the University Hospital of Messina, Messina (Italy) and written informed consent was obtained from each patient. All work was conducted in accordance with the principles of the Declaration of Helsinki.

### Nuclear Medicine procedures

**Figure 1:**
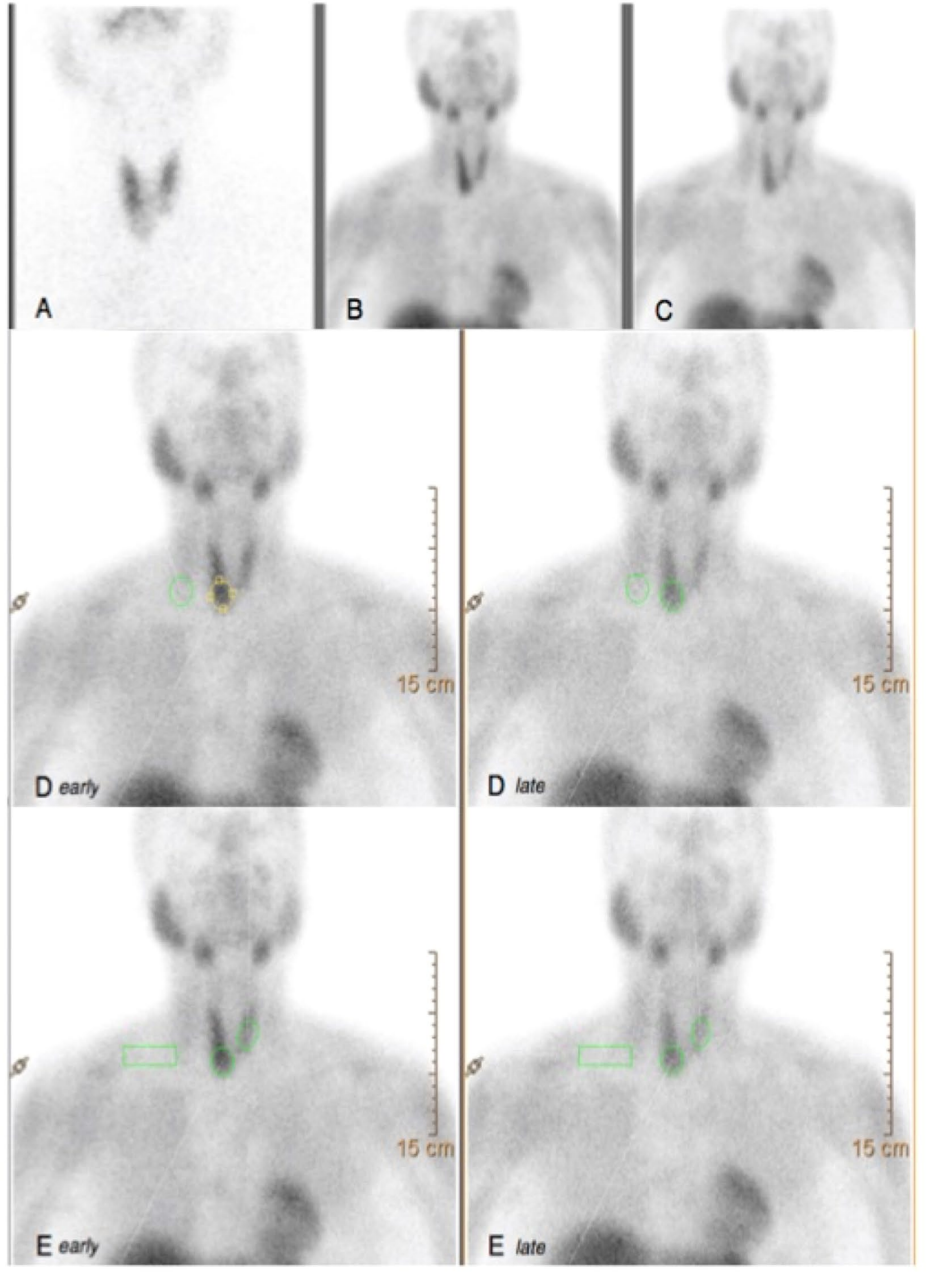
Fifty-two year-old woman with a single nodule located in the right lobe. The nodule (35 mm in maximum diameter) presented a 4B pattern at US (TI-RADS classification). (**A**) ^99m^Tc-pertechnetate (111 MBq) thyroid scintigraphy showed a well defined cold area in the lower third of the right lobe, corresponding to the US nodule. Cytopathological findings were conclusive for a class IV lesion according to the Bethesda system. (**B**,**C**) ^99m^Tc-MIBI (370 MBq) thyroid scintigraphy obtained 10 (early) and 60 (late) minutes after tracer administration. Visual MIBI analysis showed an moderate nodular tracer uptake on early image that decreased on late image. (**D**) MIBI quantitative analysis was obtained by drawing ROIs according to WO*ind* method. (**E**) MIBI quantitative analysis was obtained by drawing ROIs according to R.I. method. Calculated *WOind* and R.I. were −33% and 6.6, respectively. The patient underwent total-thyroidectomy with final histology diagnosis consistent benign non-oncocytic adenoma.

Thyroid scintigraphies were obtained by using a dual headed gamma-camera equipped with Low Energy High Resolution Parallel-hole collimators (LEHRPAR) [Brightview-X (Philips, Cleveland, USA)] and evaluated by two board certified nuclear medicine physicians with more than 20 years experience in the field. Planar anterior images (magnification: 1; matrix 256 × 256; frame time: 100 Kcounts; energy peak: 140 ± 20 KeV) of the neck were obtained 15–20 minutes after intravenous injection of ^99m^Tc-pertechnetate (74–111 MBq). Nodules were considered “cold” (i.e. hypofunctioning) if the tracer uptake was lower than in normal thyroid tissue. Additional planar images were acquired 10 and 60 minutes after intravenous administration of ^99m^Tc-MIBI (200–400 MBq) (magnification: 1 and 1.4; matrix: 256 × 256 and 128 × 128, respectively; frame time: 600 seconds; energy peak: 140 ± 20 KeV). Images were evaluated qualitatively, in double-blind modality, comparing ^99m^Tc-MIBI uptake and ^99m^Tc-pertechnetate uptake within the nodule, respectively. Visual assessment was performed using Hurtado-Lopez’s classification^16, 17^. Quantitative analysis was performed according to WO*ind* ^22^ and R.I.^19^ methods. ^99m^Tc-pertechnetate and ^99m^Tc-MIBI images (early and late) were displayed on the same screen to be more precise in drawing regions of interest (ROIs). WO*ind* was calculated by drawing an ROI around the nodule and then mirroring the ROI outside the thyroid to subtract background activity. ROIs were created on early images (+10 minutes) and successively copied on delayed ones (+60 minutes) (Fig. 1, panel D). Parameters derived from ROI analysis were: mean MIBI nodular uptake; pixel nodular number; mean MIBI background uptake; pixel background number. Then, the MIBI wash-out index (WO*ind*) was calculated as a percentage reduction value of mean MIBI nodular uptake between early (+10 minutes) and late (+60 minutes) scans. The formula employed to calculate WO*ind* is reported below:

- Mean nodular MIBI uptake (early scan) − mean background MIBI uptake (early scan) = early result (ER)
- Mean nodular MIBI uptake (late scan) − mean background MIBI uptake (late scan) = late result (LR)
- LR/ER × 100 − 100 = *wash-out index*

Immediately after, quantitative analysis was also performed according to the R.I. method^19^. On each early image (+10 minutes), an ROI was drawn around the nodule and was then mirrored onto the opposite normal thyroid lobe. These ROIs were copied onto delayed images (+60 minutes). In addition, for background subtraction, a rectangular ROI was drawn on the right superior region of the patient’s thorax and then copied onto the delayed image (Fig. 1, panel E). The mean count in each ROI was used to calculate both early ratio (ER) and delayed ratio (DR) by dividing nodule counts by normal-tissue counts after area correction for background activity. The R.I. was obtained using the formula reported below:

$${\rm{R}}.{\rm{I}}.=({\rm{DR}}-{\rm{ER}})\times 100/\mathrm{ER}.$$

All acquired data were automatically corrected according to ^99m^Tc rate decay.

As previously reported^19, 22^, nodules with RI ≥ −11.94 and WO*ind* up to −19% were rated as suspicious for malignancy on the basis of the scintigrafic criteria. Negative WO*ind* value means: early nodular MIBI uptake > late nodular MIBI uptake; negative R.I. value means: early nodular MIBI uptake > late nodular MIBI uptake; positive R.I. value means: early nodular MIBI uptake < late nodular MIBI uptake.

### Surgery and pathology

All patients underwent (near)-total thyroidectomy. Histological analyses were performed and reported by experienced endocrine pathologists.

## Results

Demographic, clinical, scintigraphic and pathological data of the patients are reported in Table 1.

**Table 1:** Demographic, Clinical, Scintigraphic and Pathological data of the 20 patients included in the study.

Patient	Sex	Age (years)	Nodule Topography	Nodule size (mm)	TI-RADS class	Bethesda system	Visual analysis pattern	R.I.	WOind (%)	Histology
1	F	20	LL	34	4A	IV	3	108.4	−10	DTC
2	F	45	RL	35	4B	III	3	75.5	−23.6	Benign Adenoma
3	F	50	RL	16	4B	IV	3	144.4	−8	DTC
4	F	80	RL	45	4B	IV	3	194.3	−6.6	DTC
5	F	54	LL	33	4A	III	2	42.7	−40	Colloid Goitre
6	M	48	RL	27	3	IV	3	93.2	−26.6	Benign Adenoma
7	F	24	RL	22	4A	III	2	27.6	−45	Colloid Goitre
8	F	33	LL	28	3	III	3	89	−24.1	Benign Adenoma
9	F	66	LL	50	3	IV	2	−24.4	−34.9	Benign Adenoma
10	M	53	RL-I	25	4A	III	2	−3.7	−21.4	Benign Adenoma
11	F	33	RL	47	3	III	2	−15.5	−23.5	Benign Adenoma
12	F	48	LL	22	4A	III	2	45	−39	Benign Adenoma
13	F	62	RL	26	3	IV	2	5.55	−25.5	Benign Adenoma
14	F	78	LL	23	4A	III	2	−70	−28.5	Benign Adenoma
15	F	52	RL	35	4B	IV	2	6.61	−33	Benign Adenoma
16	F	46	LL	27	4B	III	2	−18.6	−28	Benign Adenoma
17	F	53	LL	22	4A	III	2	12.5	−35	Benign Adenoma
18	F	75	RL	30	4B	III	3	42.5	−30	Benign Adenoma
19	M	28	I	18	4A	IV	2	48	−17	DTC
20	F	61	RL	23	4B	IV	2	40.4	−26	Benign Adenoma

LL = Left Lobe; RL = Right Lobe; I = Isthmus; TI-RADS classification: 1 = normal; 2 = benign; 3 = probably benign; 4A = undeterminated pattern; 4B = suspicious pattern; 5 = consistent with malignancy. DTC = Differentiated Thyroid Cancer.

At final histological diagnosis, four patients were affected by papillary thyroid cancer (3 follicular variant and 1 classic variant) while the remaining patients had benign lesions (2 nodular goitres and 14 benign adenomas). Hurthle cells were not found in any patient. The majority of our papillary thyroid cancer patients had a follicular variant rather than a classic variant, as expected in the context of follicular-patterned lesions at cytology. Overall, all malignant nodules were detected by WO*ind* with a threshold of −19%, while benign nodules had lower WO*ind* values. Thus, overall sensitivity, specificity, accuracy, PPV and NPV were 100% in all. On the other hand, using R.I., all malignant nodules were correctly detected (R.I. > −11.94) but 12 benign nodules (10 non-oncocytic benign adenomas) were falsely positive (60%). Finally, four patients with R.I. consistent with benign lesion (−24.4, −15.5, −70 and −18.6) had a benign adenoma at final histological diagnosis. Thus, overall sensitivity, specificity, accuracy, PPV and NPV were 100%, 57.1%, 62.5%, 25% and 100%, respectively. Results of the two methods were in agreement in forty per cent of all cases. No relevant differences were noticed between WO*ind* and R.I. results obtained taking into account or not the decay rate of ^99m^Tc.

An illustrative case is reported in Fig. 1.

## Discussion

To date, the prevalence of nodules with indeterminate cytology (i.e. AUS or SFN/FN) is not negligible, ranging from 20% to 30%. The crucial point is that the majority (up to 85%) of these patients did not have thyroid cancer at final histological diagnosis^10, 11^. Recently, in order to reduce the number of inappropriate surgeries, the American Thyroid Association (ATA)^6^ evaluated the possible role of some methods, such as molecular testing and 18F-FDG-PET/CT in differentiating malignant from benign lesions. However, the MIBI scan was not mentioned even though a few authors^5, 18, 19^ have already reported on its possible role. The very high sensitivity and NPV of a negative MIBI scan in ruling out malignancy has been reported both in prospective and in retrospective studies^5, 18^. On the contrary, a visually positive MIBI scan can be found both in malignant and benign lesions, thus reducing its specificity and PPV^5, 18^. Saggiorato and colleagues^19^ first published on the possible role of quantitative analysis (i.e. R.I. method) for improving specificity and PPV of MIBI thyroid scan in patients with follicular-patterned thyroid nodules. More recently, other Authors^7, 22^ proposed a different type of quantitative analysis, the so called WO*ind* method. In the present study, we have for the first time compared the two quantitative methods on the same series of consecutive patients in order to evaluate their diagnostic accuracy in differentiating malignant from benign lesions. The R.I. method^19^ significantly improved specificity and PPV especially among patients with non-oncocytic thyroid nodules. However, in line with our present data, false positive R.I. results (i.e. consistent with malignant lesions) were found in 2 out of 36 (5.5%) patients with non-oncocytic lesions. In our series, however, evaluating WO*ind* allowed us to correctly detect all malignant nodules without false-positive results in non-malignant ones. These differences are likely due to differences in R.I. and WO*ind* calculation: using R.I. analysis^19^, the ratio between MIBI uptake within nodules and normal tissue was obtained and compared in early and late phases. However, MIBI wash-out from normal tissue is faster compared to MIBI wash-out from nodules, both malignant and benign, as already described^23, 24^. Thus, R.I. depends significantly on MIBI wash-out from normal thyroid tissue. Accordingly, since MIBI wash-out is slower in thyroid adenomas than in colloid nodules, the value obtained in delayed image will be significantly higher than that obtained in early image, and a false positive R.I. value can occur. On the contrary, WO*ind* reproduces the trend of nodular MIBI uptake at different times without considering MIBI uptake in normal thyroid tissue. Thus, our method selectively quantifies MIBI kinetics in thyroid nodules avoiding additional variability due to MIBI kinetics in normal thyroid tissues. In addition, we think that the higher number of false positive results reported in our series using R.I. analysis compared to those already published^19^ may be due to the lower rate of malignancy (20% *vs* 41.6%, respectively). Also, the different number of patients considered in the present series compared to the previous ones^19^ may account for such difference (20 *vs* 36, respectively). Finally, the R.I. method^19^ identified a specific cut-off above which malignant nononcocytic thyroid nodules could be detected but the range of values was very wide, thus increasing the number of possible false positive results, as shown in the present comparative series. On the contrary, adopting WO*ind*, we obtained a cut-off value below which nodules could be considered suspicious for malignancy. The value range in which nodules could be considered malignant was narrower (0 to −19%) compared to that already proposed^19^, thus reducing the possibility of false positive results. This cut-off has demonstrated 100% overall sensitivity, specificity, accuracy, PPN and PPV.

## Conclusion

Quantitative MIBI scintigraphy by evaluating WO*ind* is a useful tool in the management of thyroid nodules with indeterminate cytology. Further trial in larger series of patients will be necessary before implementing MIBI quantitative analysis according to WO*ind* calculation in future guidelines on management of thyroid nodules with indeterminate cytology.
